# High glucose promotes benign prostatic hyperplasia by downregulating PDK4 expression

**DOI:** 10.1038/s41598-023-44954-2

**Published:** 2023-10-20

**Authors:** Pengyu Wei, Dongxu Lin, Changcheng Luo, Mengyang Zhang, Bolang Deng, Kai Cui, Zhong Chen

**Affiliations:** 1 Department and Institute of Urology, Tongji Hospital, Tongji Medical College, Huazhong University of Science and Technology, Wuhan, 430030 Hubei China; 2 Institute of Urology, Tongji Hospital, Tongji Medical College, Huazhong University of Science and Technology, Wuhan, 430030 Hubei China

**Keywords:** Cell biology, Molecular biology, Diseases, Pathogenesis, Urology

## Abstract

As men age, a growing number develop benign prostatic hyperplasia (BPH). According to previous research, diabetes may be a risk factor. Pyruvate dehydrogenase kinase 4 (PDK4) is closely related to glucose metabolism and plays a role in the onset and progression of numerous illnesses. This study aimed to determine the direct effects of high glucose environment on prostate epithelial cells, in particular by altering PDK4 expression levels. In this investigation, normal prostatic epithelial cells (RWPE-1) and human benign prostatic hyperplasia epithelial cells (BPH-1) were treated with 50 mM glucose to show the alteration of high glucose in prostate cells. PDK4-target siRNA, PDK4-expression plasmid were used to investigate the effects of PDK4. Rosiglitazone (RG), a PPARγ agonist, with the potential to up-regulate PDK4 expression was also used for treating prostate cells. The expression of PDK4 in human prostate samples was also analyzed. The effects of high glucose therapy on BPH-1 and RWPE-1 cells were demonstrated to enhance proliferation, epithelial-mesenchymal transition (EMT), suppress apoptosis, and down-regulate PDK4 expression. Additionally, diabetes-related BPH patients had reduced PDK4 expression. Following the application of PDK4-target siRNA, a comparable outcome was seen. The PDK4-expression plasmid therapy, however, produced the opposite results. RG with the ability to elevate PDK4 expression might be used to treat BPH. Changes in the metabolism of lipids and glucose may be the cause of these consequences. These findings showed that high glucose treatment might facilitate BPH development, and may be related to the down-regulation of PDK4. PDK4 might be a potential therapeutic target of BPH.

## Introduction

Benign prostatic hyperplasia (BPH) is a common urinary condition in elderly men, with a rising prevalence with age. By the time they reach the age of 80 or thereabouts, incidence rises to 90%^1^. Although the majority of patients are symptom-free and the progression is modest, others continue to experience severe lower urinary tract symptoms (LUTS), such as frequency, urgency, incontinence, and dysuria, which have a negative impact on patients’ quality of life^2^. Although the exact cause of BPH is unknown, several metabolic illnesses, including diabetes, obesity, hyperlipidemia, and others, are usually thought to play a role in its pathogenesis^3^. As a result, BPH is occasionally regarded as a condition linked to metabolism.

Studies from 1968 discovered a connection between diabetes and BPH^4^. In recent years, many clinical studies have shown that type 2 diabetes is associated with increased prostate volume and aggravated lower urinary tract symptoms^5,6^. In terms of mechanism, it is generally believed that it may be related to abnormal activation of the sympathetic nervous system, changes in sex hormone content, vascular atherosclerosis, and the inflammatory environment caused by diabetes^7,8^. However, the direct effect of diabetes-induced high glucose environment on prostate cells remains unclear. Yang et al.^9^ found that high glucose treatment of prostate epithelial cells can promote the process of EMT of prostate epithelial cells, and insulin can further promote this transition through the MEK/ERK pathway. However, they did not further explore the mechanism by which high glucose promotes EMT in prostate epithelial cells.

As first observed by Otto Warburg that cancer cells utilize glycolysis to metabolize glucose mainly, even in aerobic environment, which is referred to as “Warburg Effect”^10^. The metabolic reprogramming that happened in cancer had aroused wide concern among researchers. It is generally assumed that Warburg Effect ensure cancer cells could survive from hypoxic tumor microenvironment and cytotoxic effects of oxidative damage and apoptosis^11^. While as more and more researchers study this effect, it had been pointed out that some cancers with a higher level of oxidative phosphorylation (OXPHOS) are more aggressive^12^. This reminds us that, metabolic reprogramming is a complex process, based on different diseases and the state of diseases might have different manifestations.

Due to the function of secreting prostatic fluid, the normal prostate epithelial cells with unique energy metabolism^13^. And citric acid is the main component of prostatic fluid, which makes prostate epithelial cells with low OXPHOS activity, and relies on glycolysis for providing energy^14,15^. That might explain why primary prostatic cancer lacks the Warburg effect, and shows high TCA cycle/OXPHOS activity^12^.

PDK4, one of the isozymes of pyruvate kinase, has been found down-regulated in prostate cancer^12,16^. The down-regulation of PDK4 allows cancer cells with high TCA cycle/OXPHOS activity to receive more energy for proliferation^12,16^.

BPH and prostate cancer are among the most common diseases of the prostate gland. It has been reported that there with an association between them, due to them sharing some traits, however, it remains unclear what this association means^17^. That makes us curious, whether BPH has similar changes in PDK4 expression, and how these changes affect the development of BPH.

In this investigation, we sought to determine if the metabolic remodeling that high glucose causes and the role of PDK4 therein are related to the direct effects of high glucose on the prostatic epithelium.

## Methods and materials

### Cell culture

Normal prostatic epithelial cells (RWPE-1) maintained in Prostate Epithelial Cell Medium (PEpiCM), supplemented with 1% penicillin/streptomycin, 1% Prostate Epithelial Cell Growth Supplement (PEpiCGS) (ScienCell, USA). Human benign prostate hyperplasia cells (BPH-1) were cultured in RPMI 1640 medium (Gibico, USA) containing 10% FBS, 1% penicillin–streptomycin at 37 °C in a humidified 5% CO2 incubator.

### 5-ethynyl-2′-deoxyuridine (EdU) assay

The proliferation was detected by BeyoClick™ EdU cell proliferation detection kit (#C0085S, Beyotime, Shanghai, China) according to the manufacturer’s protocol. Under fluorescence microscope (200×) the nucleus was dyed blue, and EdU-positive cells were dyed red. To measure cell proliferation quantitatively, the proportion of EdU-positive cells was calculated.

### Cell counting Kit-8 (CCK-8) assay

BPH-1 and RWPE-1 cells were seeded in 96-well plates at the density of 3 × 10^3^, 1 × 10^4^ cells/well, respectively, for 24 h. After adherence, replaced the medium that contained various concentrations of Rosiglitazone (RG) and cultured for 24 h. An identical volume of DMSO served as the negative control. The cells were then treated with 10% CCK8 reagent (Yeasen Biotech Co., Ltd., Shanghai, China) for 3 h at 37 ºC. The optical density (OD) at 450 nm was then measured. Each concentration was tested in triplicate to ensure accuracy and reproducibility.

### Transient transfection

The sequences of the siRNA used to target PDK4 are as follows: sense 5′- CTACTCGGATGCTGATGAA-3′, antisense 5′-TTCATCAGCATCCGAGTAG-3′. The siRNA control sequence was provided by Guangzhou RiboBio Co., Ltd. EGFP-tagged PDK4-expression construct, GV208-PDK4-EGFP, and the EGFP-tagged empty vector construct, GV208-EGFP were provided by Shanghai Genechem Co., Ltd. PDK4-expression plasmid or siRNA were transiently transfected into the cells using Lipofectamine 3000. After transfection for 72 h, cells were collected for corresponding experiments.

### Western blot and RT-qPCR

Western blot and RT-qPCR were well established previously^18^. The primary antibodies used in this study are listed as follows: PDK4 (1:1000, 1:100; Abclone), BCL2 (1:1000; Abmart), BAX (1:1000; Abmart), Caspase3 (1:1000; Proteintech Group), E-cad (1:1000; Proteintech Group), ZO-1 (1:1000; Abmart), α-SMA (1:1000; Proteintech Group), GAPDH (1:1000; Proteintech Group), and β-tubulin (1:1000; Abmart), GAPDH and β-tubulin were used as the loading control. The original, unprocessed versions of western blots images are shown in the Supplementary Figure 1. The primers sequences used are listed in Supplementary Table 1.

### Lactate assay

Lactate production was measured using a Lactic acid assay kit (Jiancheng Bio, Nanjing, China) according to the manufacturer’s protocol. The optical density (OD) was measured at a wavelength of 530 nm and calculated according to specifications to reflect the lactate level. The relative value of the treatment group was computed after giving the control group a value of 1.

### Pyruvate assay

Pyruvate was measured using a Pyruvate assay kit (Jiancheng Bio) according to the manufacturer’s protocol. The optical density (OD) was measured at a wavelength of 505 nm and calculated according to specifications to reflect the pyruvate level. The relative value of the treatment group was computed after giving the control group a value of 1.

### Citrate assay

The relative level of citrate in cell lysates was assessed using citric acid (CA) content of the test box (Jiancheng Bio, Nanjing, China) according to the manufacturer’s protocol. Absorbance at 545 nm was measured using a microplate reader. The level of citrate in all groups was calculated and normalized to the count of cells. The control group was assigned a value of 1, and the treatment group was then calculated relative to the control group.

### Immunohistochemical staining

The patients with simple BPH and BPH complicated with T2DM were enrolled (n = 3/group). The sections of their prostate transition zone were deparaffinized in gradient xylene. Then, the sections were treated with the 3% hydrogen peroxide (H_2_O_2_) solution for 10 min to exhaust the endogenous peroxidase activity, and the sections were blocked with 10% normal goat serum for 1 h. Afterward, the sections were incubated with antibodies against PDK4 (1:100; Abclone) at 4 ℃ overnight. The sections were then incubated with biotinylated goat anti-rabbit secondary antibody (1:200; ProteinTech Group) for 30 min and reacted with 3,3-diaminobenzidine (DAB) for 10 min. At last, hematoxylin was used for counterstaining. After staining, the sections were observed under a light microscope (200 × , 400 ×).

### Statistical analysis

GraphPad Prism 8 software was used to proceed with statistical analysis. The results were expressed as mean ± standard deviation (SD) from at least three independent experiments. An unpaired student t-test was applied to compare the difference between groups.* P* < 0.05 was considered statistically significant.

### Consent for publication

All authors have read and agreed to publish this manuscript.

## Results

### High glucose promoted prostatic epithelial cells proliferation, epithelial-mesenchymal transition (EMT), and suppressed apoptosis

**Figure 1:**
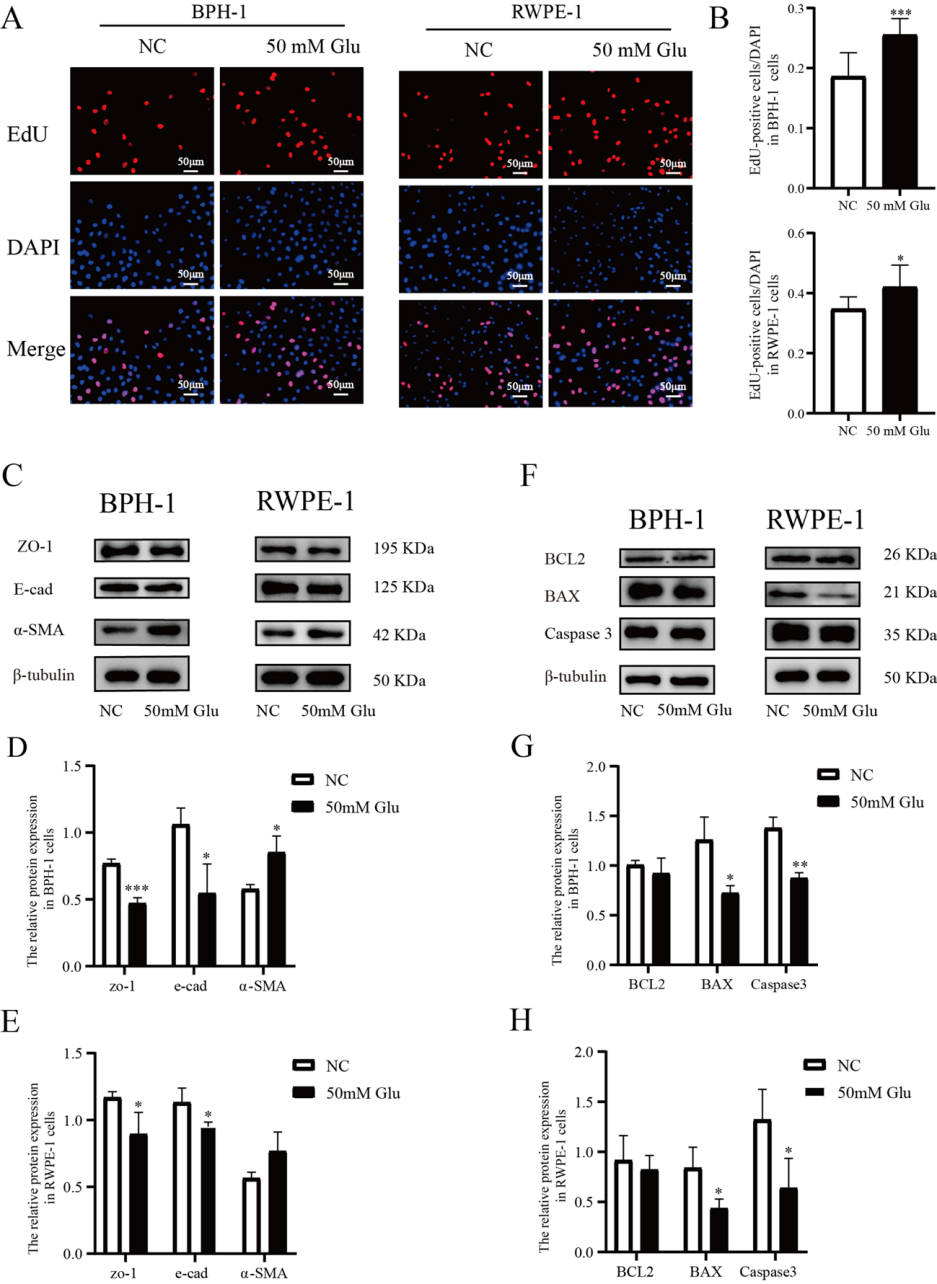
High glucose promoted prostatic epithelial cells proliferation, epithelial-mesenchymal transition (EMT) and suppressed apoptosis. BPH-1 and RWPE-1 cells were treated with ordinary culture medium or high glucose culture medium (50 mM Glu) for 24 h to evaluate the influence of the high glucose. (**A**–**B**) The cell proliferation was measured by EdU assay. (**C**–**E**) the protein expression of ZO-1, E-cad, and α-SMA calculated by western blot was used to evaluate the process of EMT. (**F**–**H**) the protein expression of BCL2, BAX, and Caspase3 was used to show the level of apoptosis. *P < 0.05; **P < 0.01; ***P < 0.001. NC = normal control, 50 mM Glu = 50 Millimoles per liter of glucose.

BPH-1 and RWPE-1 cells were treated with 50 mM glucose for 24 h to investigate the influence of high glucose on prostatic epithelial cells. The concentration of glucose was referred to others previous work^9,19^. Cell viability was determined using EdU methods. After treatment with high glucose, the proliferation of BPH-1 and RWPE-1 significantly promoted. (Fig. 1A,B) The expressions of epithelial cell markers (E-cad, ZO-1) and mesenchymal cell marker (α-SMA) were detected by western blot to show the influence of high glucose on EMT, and the significant down-regulation of E-cad and ZO-1 indicated that EMT progressed under a high glucose environment. (Fig. 1C–E) Similarly, the decreased expression level of pro-apoptotic genes (BAX, Caspase3) indicated reduced apoptosis incubating with a high glucose medium. (Fig. 1F–H).

### Down-regulation of PDK4 might participate in the high glucose regulation effects

**Figure 2:**
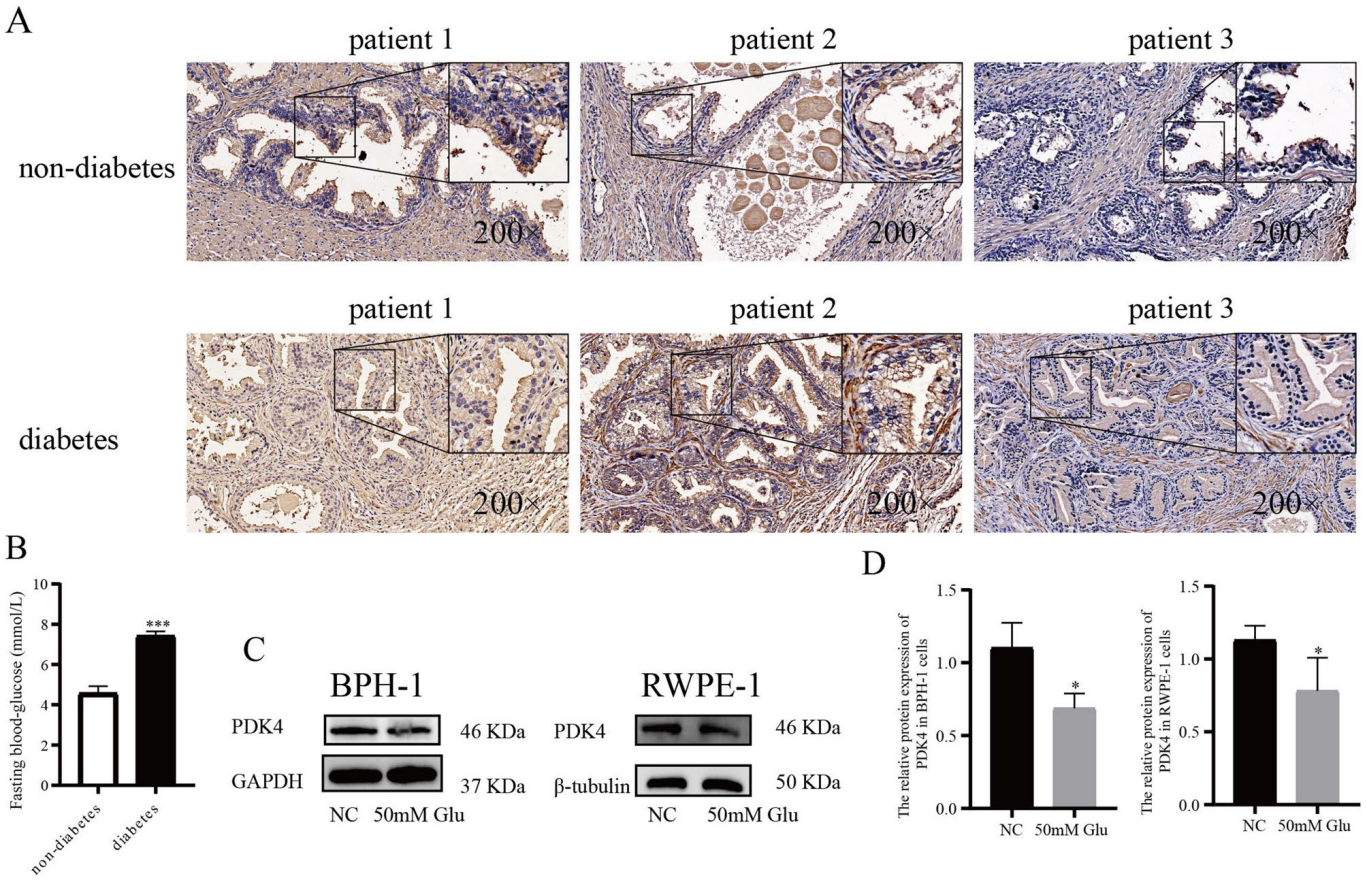
High glucose downregulation the expression of PDK4 in prostate epithelial cells. (**A**) The expression of PDK4 in prostate epithelial cells of non-diabetes and diabetes BPH patients was detected by immunohistochemical staining, then observed by optical microscope under 200 × , 400 × . (**B**) The level of fasting blood glucose in non-diabetes and diabetes BPH patients. (C-D) Western blot analysis of PDK4 expression in BPH-1, RWPE-1 cells after treating 50mM Glu 24 h. *P < 0.05; ***P < 0.001. NC = normal control, 50 mM Glu = 50 Millimoles per liter of glucose.

We examined the expression of PDK4 in non-diabetes and diabetes BPH patients (n = 3/group) prostate epithelial cells of transition zone by immunohistochemical staining, and we found that compare with non-diabetes patients, the expression of PDK4 lower in diabetes patients. (Fig. 2A,B) In vitro, the expression of PDK4 was also significantly decreased after treatment with high glucose in BPH-1 and RWPE-1 cells (Fig. 2C,D). These results indicated that the down-regulation of PDK4 might participate in the high glucose regulation effects.

### Regulated PDK4 expression in prostatic epithelial cells influence the level of proliferation, EMT, and apoptosis

**Figure 3:**
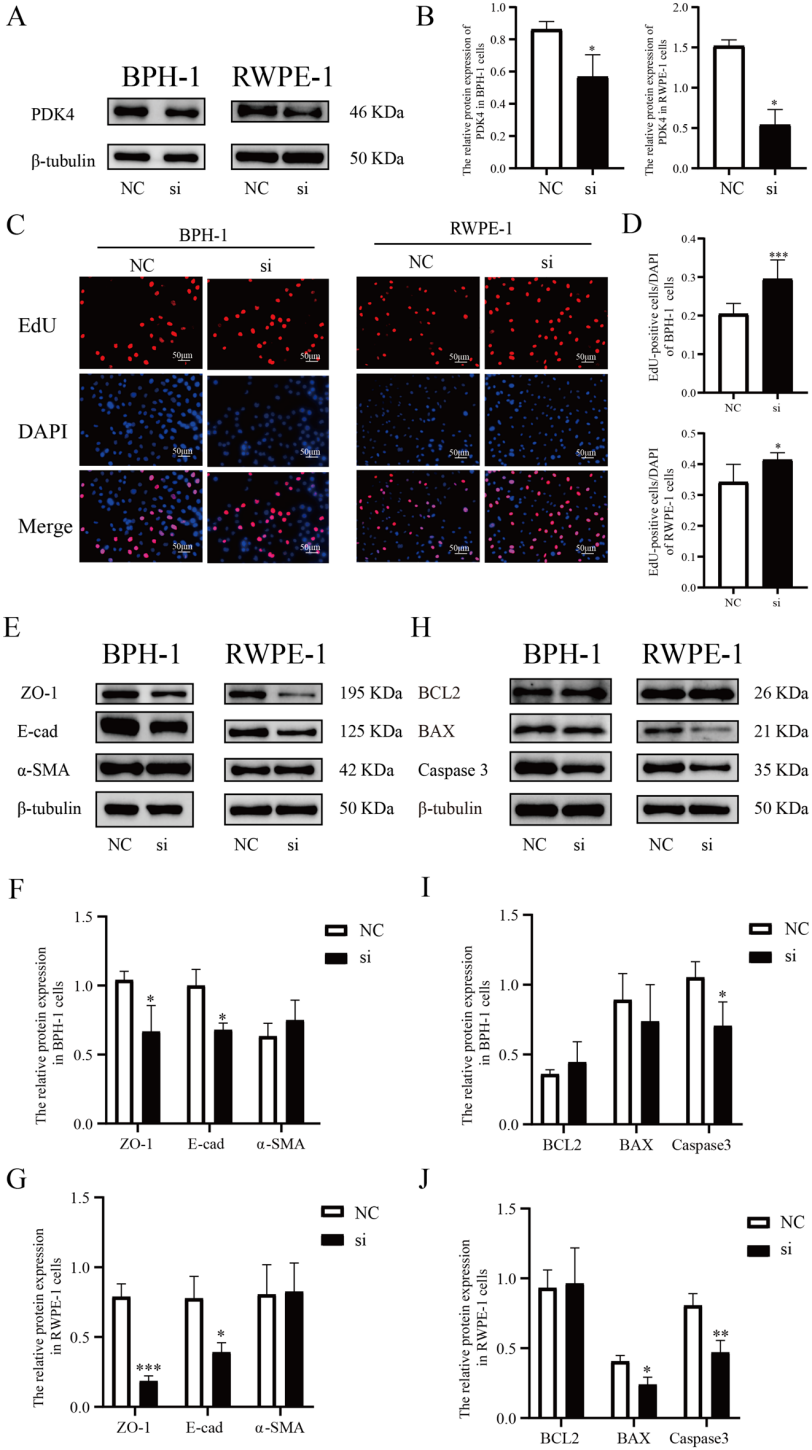
siRNA downregulation the expression of PDK4 in BPH-1, RWPE-1 cells influence the level of proliferation, EMT, and apoptosis. (**A**–**B**) The expression of PDK4 in BPH-1, RWPE-1 cells after treating with negative control or PDK4-target siRNA for 72 h. (**C**–**D**) The influence of PDK4 down-regulation on cell proliferation was measured by EdU assay. (**E**–**G**) The protein expression of ZO-1, E-cad, and α-SMA calculated by western blot was used to evaluate the influence of PDK4 down-regulation on EMT. (**H**–**J**) the protein expression of BCL2, BAX, and Caspase3 was calculated by western blot to show the influence of PDK4 down-regulation on the level of apoptosis. *P < 0.05; **P < 0.01; ***P < 0.001. NC = negative control of siRNA, si = siRNA.

We transfected PDK4-target siRNA into BPH-1 and RWPE-1 cells, and the transfection efficiency was confirmed by western blot after transfecting for 72 h (Fig. 3A,B). Then the proliferation was determined using EdU methods. The results showed that after transfecting for 72 h, the down-regulation of PDK4 significantly promoted the proliferation of BPH-1 and RWPE-1 cells (Fig. 3C,D). Besides proliferation, the influence on EMT and apoptosis were detected by western blot. The siRNA treatment significantly decreased the expression of E-cad, ZO-1, BAX, and Caspase3, which indicated the progression of EMT (Fig. 3E–G) and inhibited apoptosis (Fig. 3H–J).

**Figure 4:**
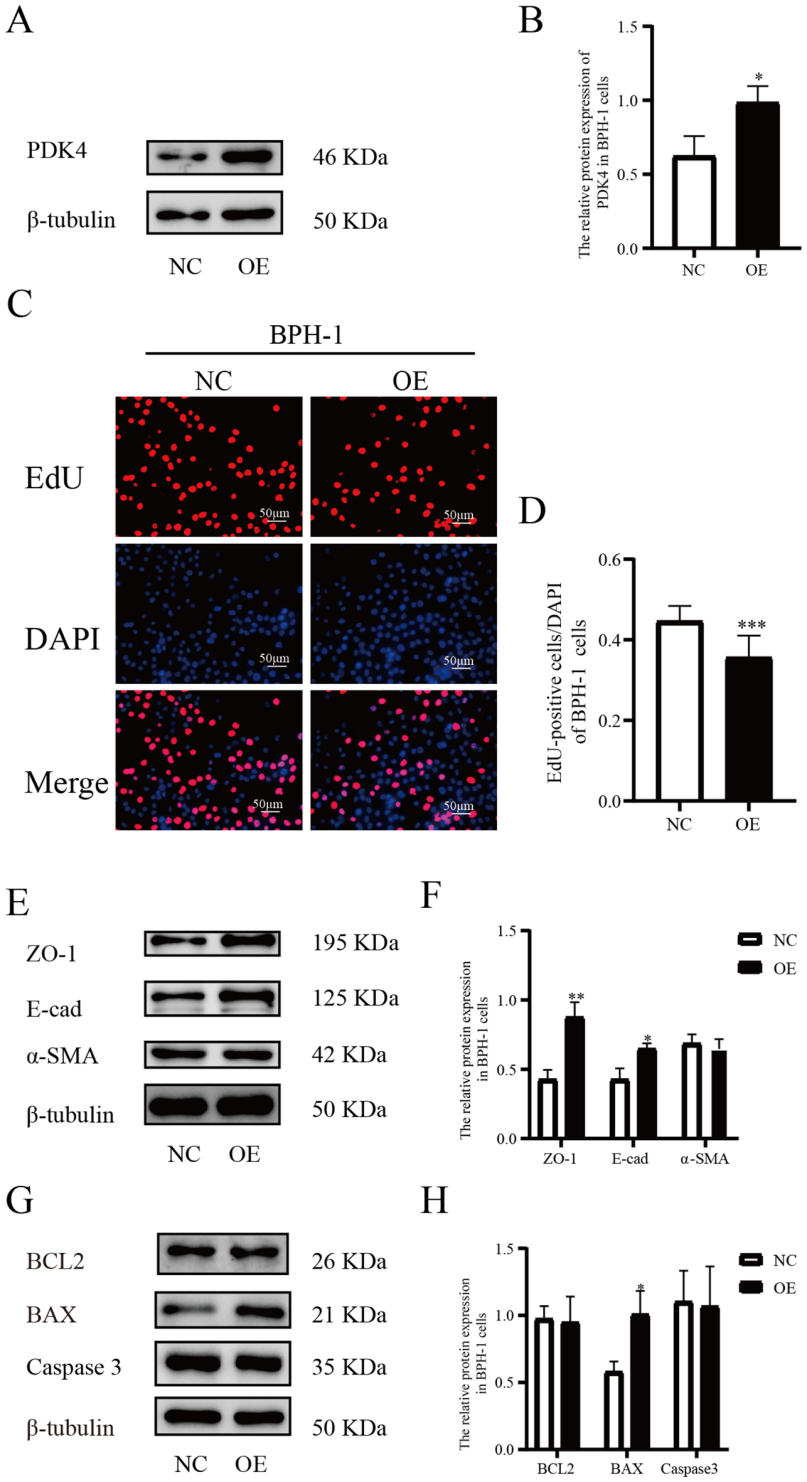
PDK4-expression plasmid upregulated the expression of PDK4 in BPH-1 cells influencing the level of proliferation, EMT, and apoptosis. (**A**–**B**) The expression of PDK4 in BPH-1 cells after treating with negative control or PDK4-expression plasmid for 72 h. (**C**–**D**) The influence of PDK4 up-regulation on cellration was measured by EdU assay. (**E**–**F**) The protein expression of ZO-1, E-cad, and α-SMA calculated by western blot was used to evaluate the influence of PDK4 up-regulation on EMT. (**G**–**H**) the protein expression of BCL2, BAX, and Caspase3 was calculated by western blot to show the influence of PDK4 up-regulation on the level of apoptosis. *P < 0.05; **P < 0.01; ***P < 0.001. NC = negative control of plasmid, OE = overexpression plasmid.

To further confirm the effects of PDK4 on prostatic epithelial cells, we transfected the PDK4-expression plasmid into BPH-1 cells, and the transfection efficiency was confirmed by western blot after transfected for 72 h (Fig. 4A,B). Then we repeated the experiments mentioned above, and found that overexpression of PDK4 in prostatic hyperplastic epithelial cells could suppress proliferation (Fig. 4C,D), inhibit EMT (Fig. 4E–F), and induce apoptosis (Fig. 4G–H).

### RG could increase the expression of PDK4 in prostatic epithelial cells and with the ability to influence the level of proliferation, EMT, and apoptosis

**Figure 5:**
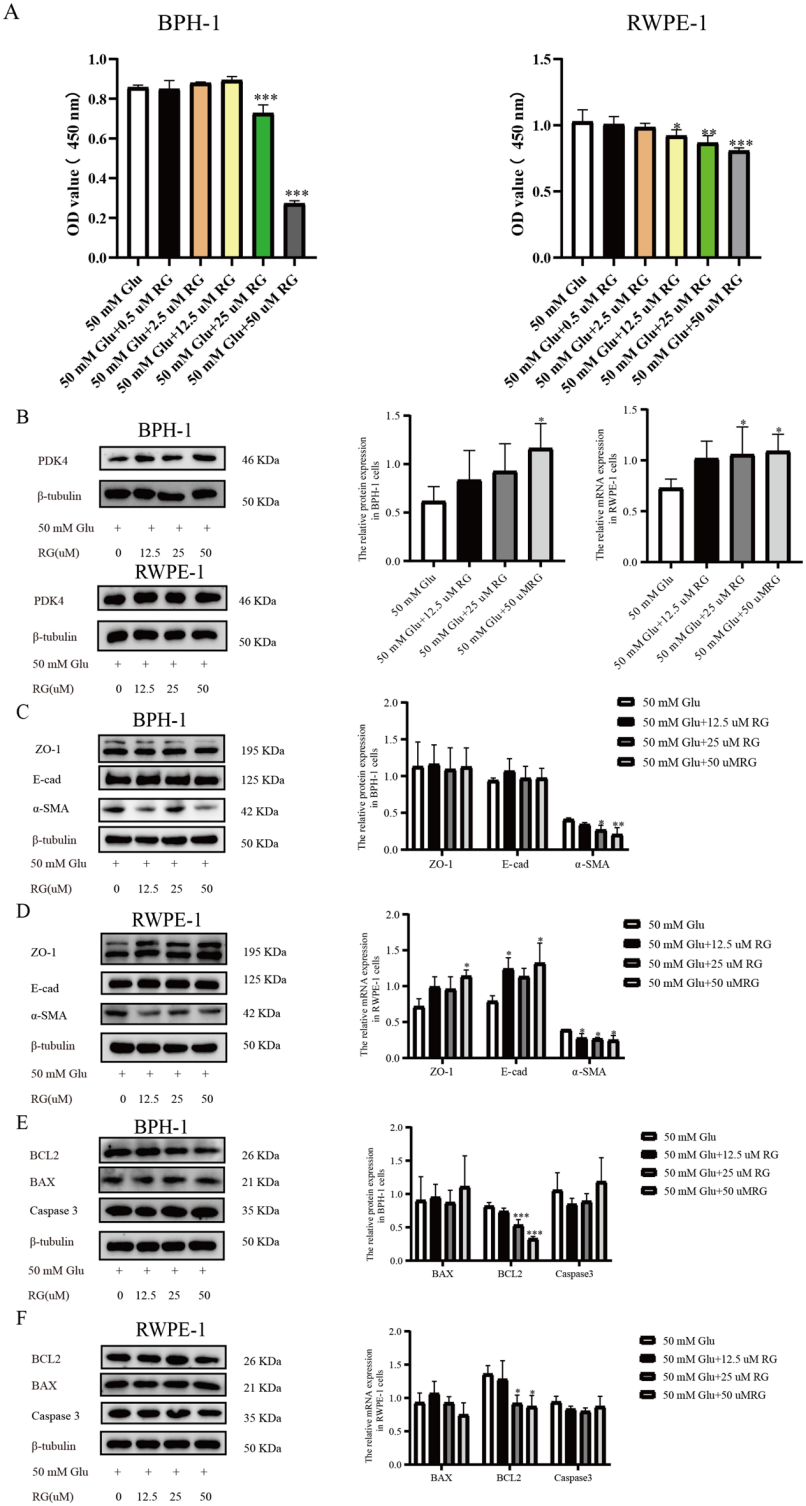
RG could increase the expression of PDK4 in prostatic epithelial cells and with the ability to influence the level of proliferation, EMT, and apoptosis. (**A**) The value of OD 450 nm in BPH-1 and RWPE-1 cells after treating with 50 mM Glu and different concentrations of RG for 24 h. (**B**) The expression of PDK4 in BPH-1 and RWPE-1 cells after treating with different concentrations of RG for 24 h. (**C**–**D**) The protein expression of ZO-1, E-cad, and α-SMA calculated by western blot was used to evaluate the influence of RG on EMT. (**E**–**F**) the protein expression of BCL2, BAX, and Caspase3 was calculated by western blot to show the influence of RG on the level of apoptosis. *P < 0.05; **P < 0.01; ***P < 0.001. RG = Rosiglitazone, 50 mM Glu = 50 Millimoles per liter of glucose.

Rosiglitazone (RG), the agonist of PPARγ and a common hypoglycemic drug, has been reported to increase the level of PDK4^20,21^. To determine the impact of RG on the therapy of BPH, we subject BPH-1 and RWPE-1 cells to a variety of RG concentrations and 50 mM Glu. The CCK8 assay results demonstrated that RG could reduce the viability of BPH-1 and RWPE-1 cells after treatment for 24 h, and that this effect was positively linked with concentration (Fig. 5A). Through the application of the western blot, we discovered that RG could enhance PDK4 expression in BPH-1 and RWPE-1 cells (Fig. 5B). The effect of RG on EMT and apoptosis were also demonstrated by western blot. RG treatment significantly decreased the expression of α-SMA in BPH-1 cells and decreased the expression of α-SMA, increased the expression of E-cad and ZO-1 in RWPE-1 cells which indicated that RG could suppress the progression of EMT (Fig. 5C–D). And the down-regulation of BCL2 in BPH-1 and RWPE-1 cells indicated that RG could enhance apoptosis (Fig. 5E–F).

### The glucose and lipid metabolism affected by high glucose and PDK4 regulation in prostatic epithelial cells

**Figure 6:**
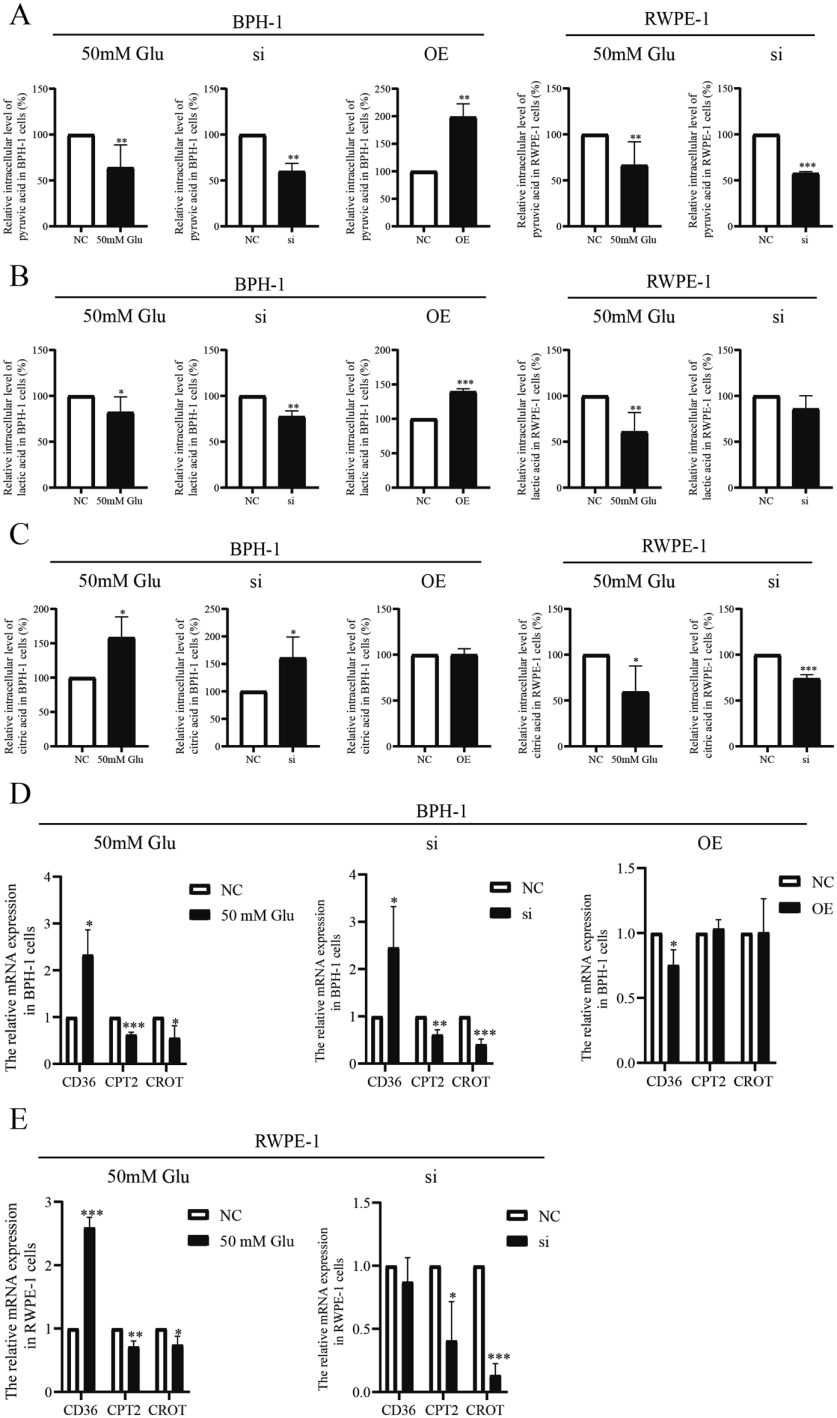
The glucose and lipid metabolism is affected by high glucose and PDK4 regulation in prostatic epithelial cells. After BPH-1 cells treated with high glucose for 24 h, PDK4-target SiRNA, PDK4-expression plasmid for 72 h, RWPE-1 cells treated with high glucose for 24 h, PDK4-target SiRNA for 72 h, the intracellular level of pyruvic acid (**A**), lactic acid (**B**), citric acid (**C**) was calculated by relative Assay Kit to show the influence of glucose metabolism under corresponding treatment; the mRNA expression of CD36, CPT2, CROT (**D**–**E**) was calculated by RT-PCR to show the influence of lipid metabolism under corresponding treatment. *P < 0.05; **P < 0.001; ***P < 0.001.

To verify the underlying mechanism of high glucose and PDK4 regulation in prostatic epithelial cells, the level of glucose and lipid metabolism were examined. High glucose and PDK4-target siRNA treatment showed decreased pyruvate and lactate content intracellular in BPH-1 and RWPE-1 cells. While the content of citrate increased in BPH-1 cells and decreased in RWPE-1 cells. These changes indicated indirectly that high glucose might down-regulate PDK4 expression, consequently, promote cellular aerobic glucose metabolism. The different changes in citrate content might due to the differences in the ability to handle citric acid. However, PDK4-expression plasmid increased pyruvate and lactate content, decreased citrate content intracellular in BPH-1 (Fig. 6A–C). This further confirmed that PDK4 plays a crucial role in the regulation of prostatic epithelial cells glucose metabolism.

We also examined several key enzymes in glucose and lipid metabolism by RT-PCR. Carnitine Octanoyltransferase (CROT) catalyzes the reversible transfer of fatty acyl groups between CoA and carnitine, palmitoyltransferase II (CPT2) catalyzes the transferring of long-chain fatty acids, they are the key enzymes of β-oxidation of fatty acids. Results showed that the expression of CROT and CPT2 significantly down-regulated after high glucose and siRNA treatment, which suggested that the β-oxidation of fatty acids be inhibited^22,23^. CD36 significantly up-regulated, which indicated the transportation of fatty acids had been facilitated^24^. However, PDK4-overexpression exhibited the opposite trend (Fig. 6D–E).

Taken together, these results indicated that PDK4 regulates glucose and lipid metabolism and may contribute to the progression of BPH under high glucose solution.

## Discussion

The present study demonstrated that high glucose treatment could induce proliferation and EMT, and suppress apoptosis in prostatic epithelial cells. These effects might relate to the downregulation of PDK4.

BPH, is one of the most common prostate diseases in old-age males, with the annoying lower urinary tract symptoms (LUTS) significantly disturbing patients’ quality of life. The precise etiology of BPH needs further exploration, however, the association between BPH and diabetes has been reported by numerous studies^8,25^. However, the direct influence of high glucose in the prostate remains insufficient. Ye et al.^19,26^ found that under a high glucose environment, the level of reactive oxygen species (ROS) significantly up-regulated, which induced the proliferation, and suppressed the apoptosis of prostate epithelial cells. In this study, we confirmed that high glucose could affect the proliferation and apoptosis level by EdU and western blot experiment. In the meantime, we demonstrated that high glucose could accelerate the process of EMT, which is in conformity with the results of Yang et al.^9^.

PDK4 is one of the pyruvate dehydrogenase kinase family isoenzymes, involved in glucose metabolism, it inhibits oxidative phosphorylation and affects energy production by phosphorylating pyruvate dehydrogenase complex (PDC)^20^. As the major nutrient of glucose metabolism, the level of glucose could regulate the expression of PDK4^20^. Additionally, PDK4 can affect substance metabolism, and the decrease of PDK4 can promote fatty acid synthesis and amino acid synthesis^27^. The increased expression of PDK4 served as a characteristic of the Warburg effect, and had been found with oncogenic effects in several cancers^28,29^. However, the expression of PDK4 in some cancers, which benefit from a high level of OXPHOS activity is down-regulated^30–32^. Particularly, consistent with the lacking the Warburg effect, it had been reported that the expression of PDK4 is significantly down-regulated in prostate cancer and is related to tumor recurrence, and drug resistance^16,33^.

Beyond that, it had been reported the overexpression of PDK4 could restrain EMT induced by TGFβ^33^. Which had been proven with significant importance in BPH development^34^. Based on these previous researches we surmised that the down-regulation of PDK4 has a role in the development of BPH.

To explore the possible mechanism of how high glucose affects prostate epithelial cells, we examined the expression of PDK4 in non-diabetes, diabetes BPH patients’ prostates, and in high glucose treated BPH-1 and RWPE-1 cells. The results indicated that high glucose might decrease the expression of PDK4. This led us to further explore the effects of the downregulation of PDK4 on BPH progression.

As a proliferative disease, abnormal dynamic proliferation and limited apoptosis are the essential characteristics of BPH^35^. By transfection of PDK4-target siRNA into BPH-1, RWPE-1 cells we found that the EdU positive cells increased, which indicated a stronger proliferation ability. Meanwhile, the level of apoptosis was decreased, as the expression of pro-apoptosis genes (BAX, Caspase3) decreased. These results supported that the downregulation of PDK4 could stimulate BPH development.

The decreasing of epithelial cell markers such as E-cad and ZO-1, and the increasing of mesenchymal cell markers such as N-cadherin and α-SMA are generally referred to as EMT^36^. Upon the progression of EMT, the mesenchymal-like cells increased which with stronger proliferation and resisted apoptosis^37^. Recent findings indicated that the change in glycolysis and OXPHOS are intertwined with EMT^38^. PDK4 as a key enzyme of glycolysis, had been reported with the ability to adjust EMT^33^. In this study, we found that the treatment of PDK4-target SiRNA could decrease the expression of E-cad and ZO-1.

To further confirmed the effects of PDK4 regulation on BPH progression and the possible therapeutic effects, we transfected the PDK4 expression plasmid into the BPH-1 cells. The results showed that overexpression of PDK4 in the prostatic hyperplasia cell line could suppress the progression of BPH, manifested as a lower level of proliferation, higher level of apoptosis, and limited EMT. This means drugs target PDK4 might with the potential for the treatment of BPH. And we discovered that RG with the potential to upregulate PDK4 might decrease prostate proliferation and EMT while promoting apoptosis.

Besides the common features of BPH, the content of pyruvic acid, lactic acid, and citric acid was measured tried to have a better understanding of the direct influence of high glucose and PDK4 regulation on BPH development. Our results showed a decreased level of pyruvic acid and lactic acid under high glucose or siRNA treatment, which might indicate a reduction of anaerobic glycolysis and an increase in the TCA cycle. The treatment of PDK4-expression plasmid showed opposite changes, which further confirmed the effects of PDK4 on glucose metabolism. However, changes in the citric acid in the two cell lines are different, which may be due to the different enzymatic activity of the TCA cycle. Since PDK4 is involved in the regulation of fatty metabolism^26^, we also measured the expression of mRNA of key enzymes of lipid metabolism. The PCR showed, the expression of CD36 was significantly up-regulated under high glucose or siRNA treatment, and significantly down-regulated under PDK4-expression plasmid treatment. CD36 is a scavenger receptor expressed in cell membranes that participate in lipid uptake through binds to diverse ligands that could accelerate cells proliferation^39^. These results indicated that the influence of PDK4 in BPH progression might be related to the adjustment of glucose and lipid metabolism.

In this study, we show that the down-regulation of PDK4 might involve in the high glucose effects in BPH development for the first time. However, the specific mechanism had only been briefly discussed, further experiments are needed to show the overall metabolic changes and signal pathways that are related to these effects.

## Conclusion

High glucose treatment could accelerate prostate epithelial cells proliferation, EMT, and suppress apoptosis. The down-regulation of PDK4 might be involved in these effects.

### Supplementary Information

Supplementary Figure 1.

Supplementary Table 1.
