# Supplementary material for: High glucose promotes benign prostatic hyperplasia by downregulating PDK4 expression
Source: Sci Rep. 2023 Oct 20;13:17910. doi: 10.1038/s41598-023-44954-2 (PMC10589318; doi:10.1038/s41598-023-44954-2)
Supplement: Supplementary file 1 — Supplementary Figure 1. [file 41598_2023_44954_MOESM1_ESM.docx]

**Supplementary Figures**

“High glucose promotes benign prostatic hyperplasia by downregulating PDK4 expression”

Pengyu Wei^1,2^, Dongxu Lin^1,2^, Changcheng Luo^1,2^, Mengyang Zhang^1,2^, Bolang Deng^1,2^, Kai Cui^1,2^, Zhong Chen^1,2*^

*^1^Department of Urology, Tongji Hospital, Tongji Medical College, Huazhong University of Science and Technology, Wuhan 430030, Hubei, China;*

*^2^Institute of Urology, Tongji Hospital, Tongji Medical College, Huazhong University of Science and Technology, Wuhan 430030, Hubei, China;*

*Corresponding author: Zhong Chen, Department and Institute of Urology, Tongji Hospital, Tongji Medical College, Huazhong University of Science and Technology, Wuhan 430030, Hubei, China.


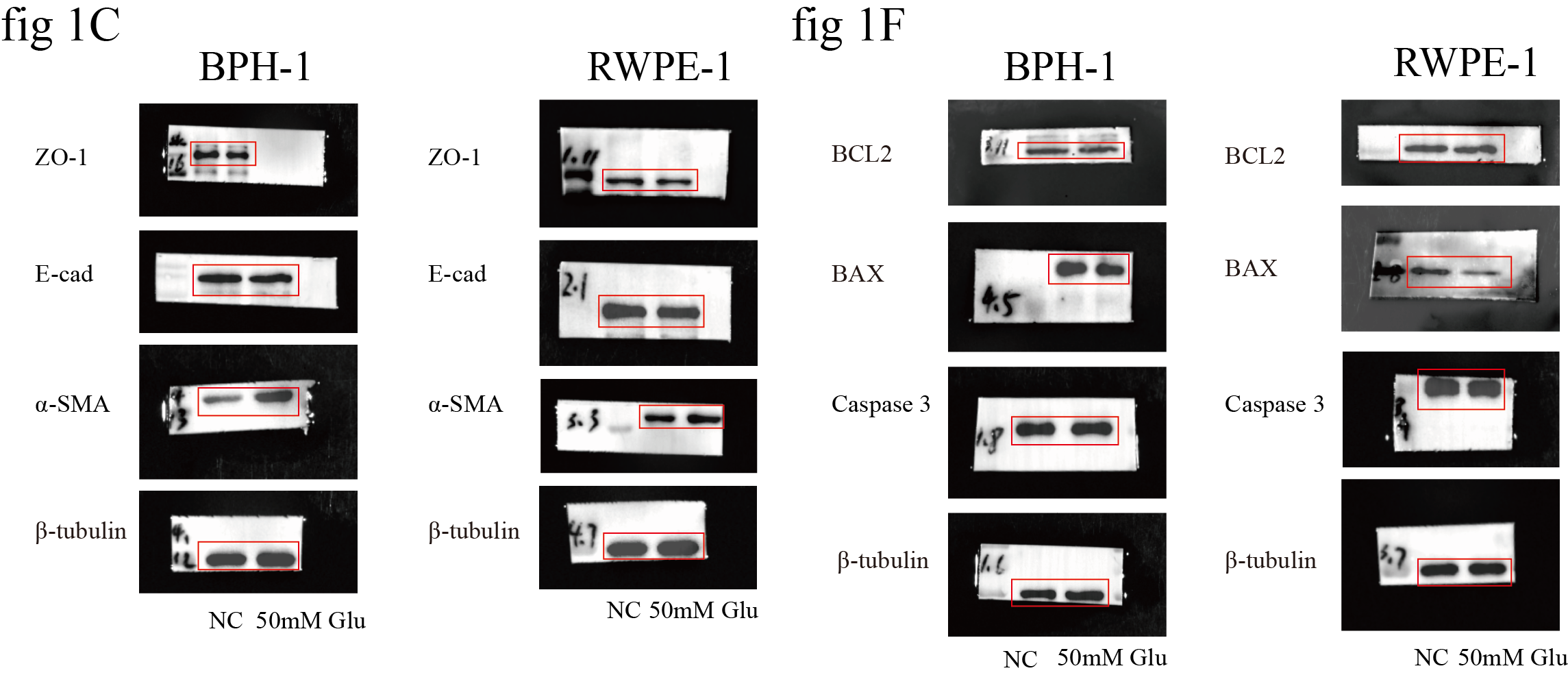


Supplementary Figure S1. Uncropped original images of Western blots in Fig. 1.


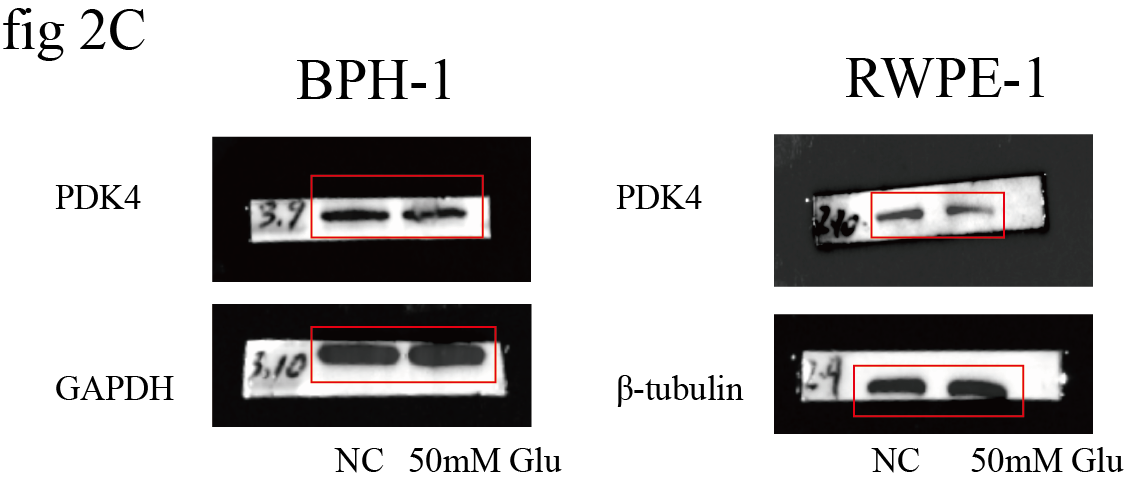


Supplementary Figure S2. Uncropped original images of Western blots in Fig. 2.


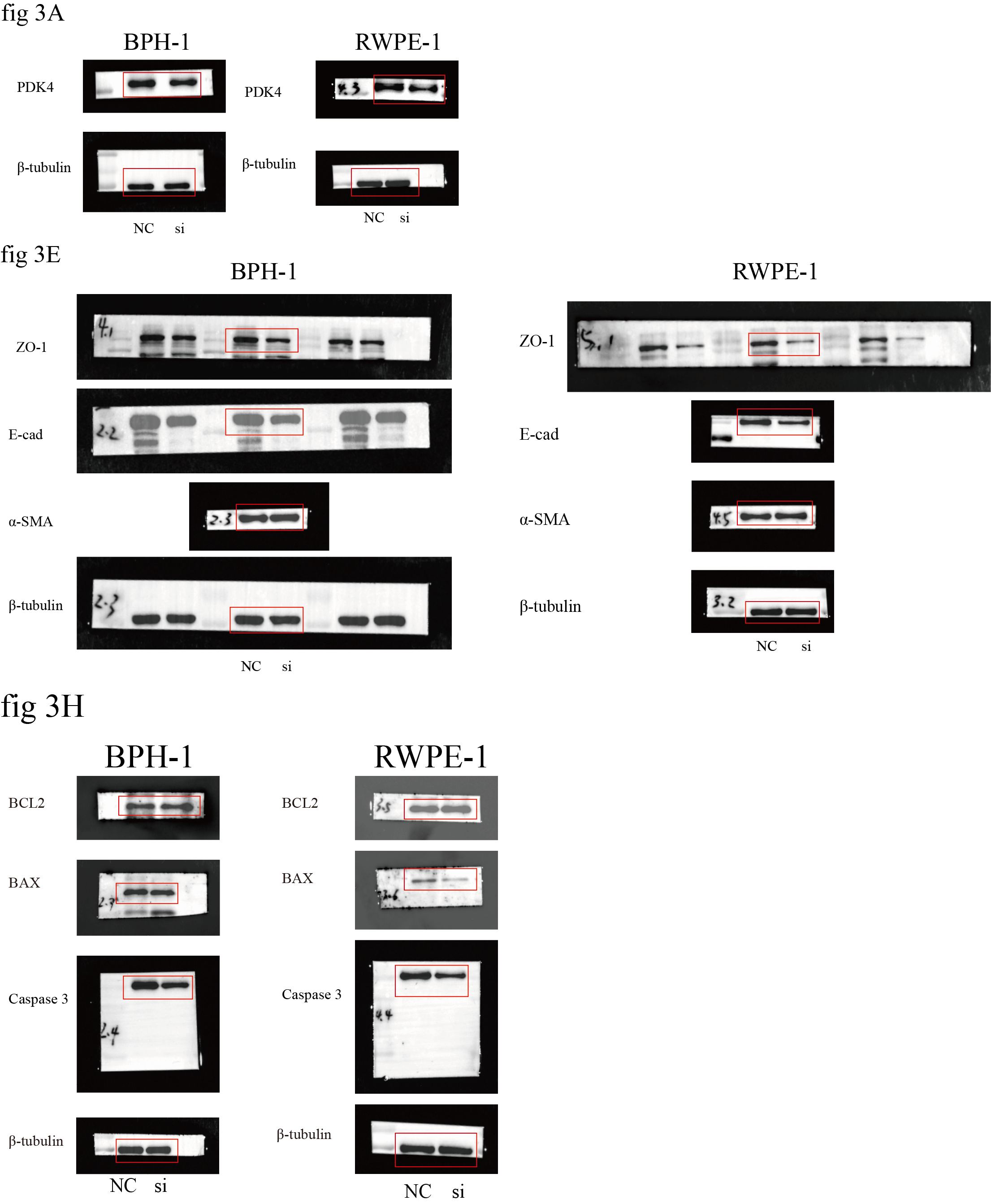


Supplementary Figure S3. Uncropped original images of Western blots in Fig. 3.


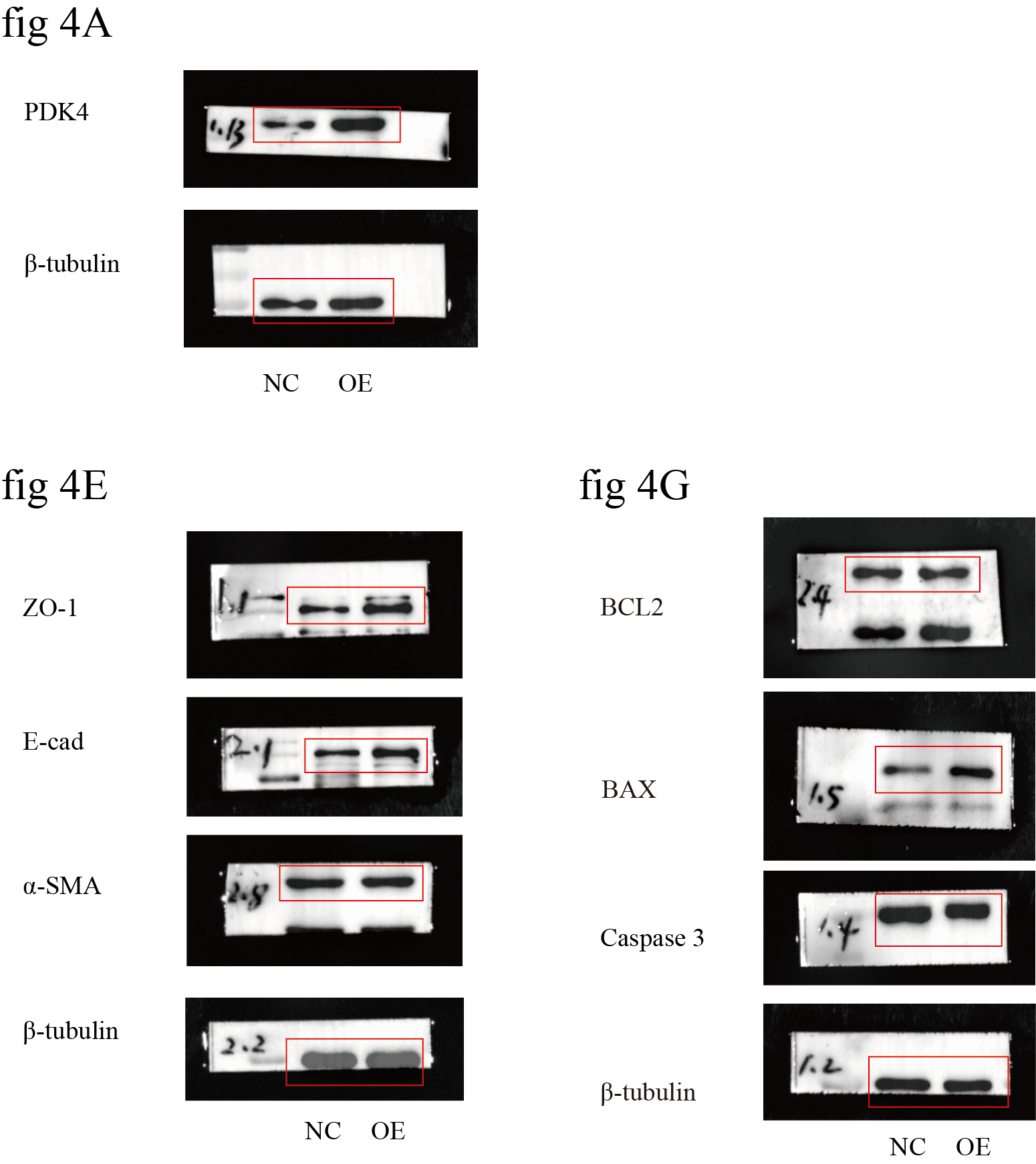


Supplementary Figure S4. Uncropped original images of Western blots in Fig. 4.


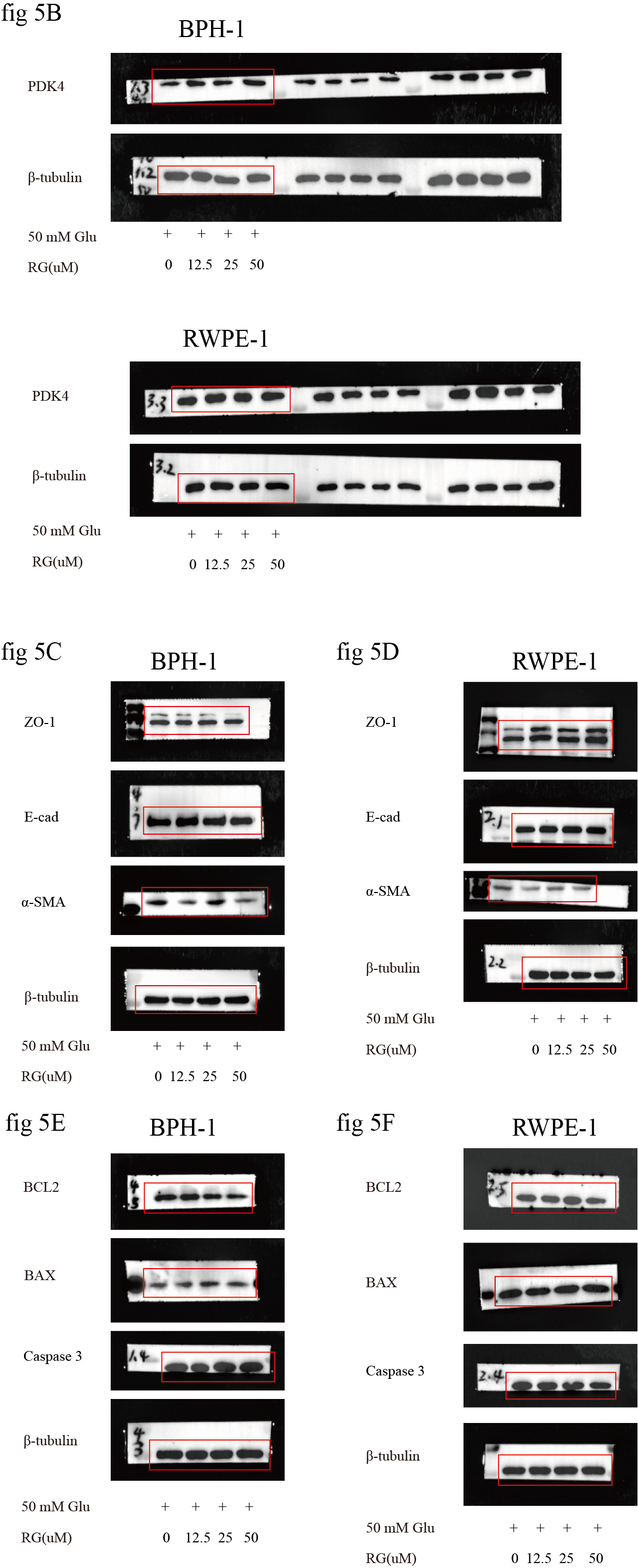


Supplementary Figure S5. Uncropped original images of Western blots in Fig. 5.
