# Supplementary material for: High glucose promotes benign prostatic hyperplasia by downregulating PDK4 expression
Source: Sci Rep. 2023 Oct 20;13:17910. doi: 10.1038/s41598-023-44954-2 (PMC10589318; doi:10.1038/s41598-023-44954-2)
Supplement: Supplementary file 2 — Supplementary Table 1. [file 41598_2023_44954_MOESM2_ESM.docx]

Supplementary Table1. The list and sequence of long primers used for RT-qPCR analysis

| Gene | Sequence (5’-3’) | Length (bases) |
| --- | --- | --- |
| β-actin |  |  |
| F-primer | GCGCGGCTACAGCTTCA | 17 |
| R-primer | CTTAATGTCACGCACGATTTCC | 22 |
| CD36 |  |  |
| F-primer | GGCTGTGACCGGAACTGTG | 19 |
| R-primer | AGGTCTCCAACTGGCATTAGAA | 22 |
| CROT |  |  |
| F-primer | GTGGTGGCTGAATGTTGCCTA | 21 |
| R-primer | TTGGAGGCCAGTAGTGTTCAA | 21 |
| CPT2 |  |  |
| F-primer | CATACAAGCTACATTTCGGGACC | 23 |
| R-primer | AGCCCGGAGTGTCTTCAGAA | 20 |
| G6PC1 |  |  |
| F-primer | GTGTCCGTGATCGCAGACC | 19 |
| R-primer | GACGAGGTTGAGCCAGTCTC | 20 |
| PDK4 |  |  |
| F-primer | AGAGGTGGAGCATTTCTCGC | 20 |
| R-primer | ATGTTGGCGAGTCTCACAGG | 20 |
